# The Evasion of Antiviral Innate Immunity by Chicken DNA Viruses

**DOI:** 10.3389/fmicb.2021.771292

**Published:** 2021-10-28

**Authors:** Li Gao, Shijun Zheng, Yongqiang Wang

**Affiliations:** Department of Preventive Veterinary Medicine, College of Veterinary Medicine, China Agricultural University, Beijing, China

**Keywords:** chicken, DNA virus, PRRs, innate immunity, evasion

## Abstract

The innate immune system constitutes the first line of host defense. Viruses have evolved multiple mechanisms to escape host immune surveillance, which has been explored extensively for human DNA viruses. There is growing evidence showing the interaction between avian DNA viruses and the host innate immune system. In this review, we will survey the present knowledge of chicken DNA viruses, then describe the functions of DNA sensors in avian innate immunity, and finally discuss recent progresses in chicken DNA virus evasion from host innate immune responses.

## Introduction

The innate immune system constitutes the first line of host defense. Upon virus infection, pathogen-associated molecular patterns (PAMPs), which are the components of foreign pathogens, will be recognized by germline-encoded pattern-recognition receptors (PRRs), leading to the initiation of innate antiviral immunity, such as the production of type I interferons (IFN-I) and pro-inflammatory cytokines. On the other hand, viruses have evolved multiple strategies to evade host immune surveillance which has been explored extensively for human viruses. There is growing evidence indicating the interaction between avian DNA viruses and the host’s antiviral innate immunity. This review will focus on chicken DNA viruses and their evasion of host innate immune response, surveying the present knowledge of the function of DNA sensors in avian innate immunity, and discussing futuristic perspectives in this angle. Monumental reviews specific on DNA viruses and antiviral innate immunity have been summarized (Briard et al., 2020).

## The Mainly Circulated Chicken DNA Virus

Multiple DNA viruses threaten the poultry industry, including chicken infectious anemia virus (CIAV), Fowl adenovirus serotype 4 (FAdV-4), Marek’s disease virus (MDV), infectious laryngotracheitis virus (ILTV), and fowl pox virus (FPV).

CIAV is a non-enveloped, single-stranded DNA virus, which belongs to the *Gyrovirus* genus, the *Anelloviridae* family. The genome of CIAV is a 2.3kb covalently linked DNA ring coding for three viral proteins (Li et al., 2016). CIAV mainly infects bone marrow hemocytoblasts and thymus precursor lymphocytes (Miller et al., 2005). The severe damages caused by this virus such as weight loss, anemia, intramuscular hemorrhage, lymphoid atrophy, and bone marrow aplasia mostly appear among chicks less than 2-week-old and are void of maternally derived antibodies (Gimeno and Schat, 2018). Adult chickens are also infected horizontally with CIAV, though with subclinical symptoms after the maternal antibodies wane (Trinh et al., 2015; Fatoba and Adeleke, 2019).

CIAV infection causes chicken immunosuppression. Inoculation of 1-day-old chicks with CIAV suppresses functions of spleen and bone-marrow-derived macrophage, such as Fc receptor expression, phagocytosis, and IL-1 production (McConnell et al., 1993). Four days post inoculation of 1-day-old chicks with CIAV, the innate immune cytokine mRNA levels were analyzed using qRT-PCR and gene expression microarrays, the pro-inflammatory cytokines, including IL-1β, IL-6, CXCLi2, and IFN-I, were inhibited across different tissues, except in the thymus (Giotis et al., 2015). Compared to the uninfected control thymus, CIAV infection-induced IL-1β, IL-6, and IFN-I production by 3–4 folds. However, that is limited in magnitude. Not all of the pro-inflammatory cytokines could be induced by CIAV infection. For example, CXCLi2 was significantly inhibited in the bursa of the Fabricius at 11days post-inoculation (Giotis et al., 2015). CIAV infection of MSB1 cells, a T cell line that supports CIAV replication *in vitro*, cannot induce an effective antiviral innate immune response either. When MSB-1 cells were infected with CIAV, IFN-I and interferon-stimulated genes (ISGs) mRNAs were only slightly induced at 24 and 48hpi, and even declined at 72hpi compared with uninfected control cells (Giotis et al., 2015, 2018).

FAdV belongs to the genus *Aviadenovirus* and the family *Adenoviridae*. FAdV can be divided into five species (FAdV-A to FAdV-E) according to their restriction enzyme digestion pattern (Zsak and Kisary, 1984; Li et al., 2017) and 12 serotypes (FAdV-1–8a and 8b–11) based on cross-neutralization test (Hess, 2000; Kim et al., 2014). FAdV-4, one of 12 FAdV serotypes, has been grouped into FAdV-C (Li et al., 2017; Pan et al., 2017). The genome of FAdV-4 is an ~43–46kb double-stranded DNA, encoding for 10 major structural proteins in the viral particles and 11 non-structural proteins, which participate in viral replication and pathogenesis (Li et al., 2017). FAdV-4 mainly infects chicken livers and can cause severe damage in chickens, especially for broilers aged 3- to 5-week-old. The mortality rate caused by FAdV-4 infection is about 30–70% (Mansoor et al., 2011; Shah et al., 2012; Zhao et al., 2015; Shah et al., 2016). FAdV-4 infection in chickens can induce hydropericardium syndrome (HPS), which is a severe disease characterized by the accumulation of clear, straw-colored fluid in the pericardial sac, as well as nephritis and hepatitis (Abdul-Aziz and Al-Attar, 1991; Abdul-Aziz and Hasan, 1995; Abe et al., 1998).

A previous review has summarized that human adenovirus (HAdV) inhibits innate host immunity through its early gene products and is implicated in pathogenesis (Wang and Zhao, 2019). For example, the E1A oncogene of HAdV-5 blocks the cellular response to IFNs (Ackrill et al., 1991) and attenuates the transcription of IL-6 (Janaswami et al., 1992). Hyper-virulent FAdV-4 also suppresses host immune response. Compared to the mock-infected control, FAdV-4 infection depleted lymphocytes in the thymus and bursa through apoptosis and severe inflammatory response (Niu et al., 2019b). Analysis of the mRNA expression in livers of FAdV-4-infected chickens identified that IFN was downregulated at 7days post-infection (Ren et al., 2019). However, the mechanisms of how FAdV-4 suppresses host innate immune response remain to be explored.

Marek’s disease virus (MDV) is a member of the genus *Mardivirus*, which belongs to the subfamily *Alphaherpesvirinae* in the order of *Herpesvirales* (Gimeno and Schat, 2018). Despite the use of vaccines, MDV infection still circulates in poultry flocks. It mainly infects chickens, causing lymphomas and different kinds of nonneoplastic syndromes, including neurological syndromes, lymphodegenerative syndrome, and atherosclerosis (Gimeno and Schat, 2018; Bertzbach et al., 2020).

As an immunosuppressive virus, MDV infection inhibits the host’s innate immune responses through multiple approaches. RT-PCR and ELISA analysis suggested that, IFN-I mRNA and protein expressions were significantly down-regulated in the thymus and bursa of MDV-infected groups (Sun et al., 2019). Furthermore, the potential of MDV inhibition on IFN-I was associated with their pathogenesis (Sun et al., 2019). Macrophages and Natural killer (NK) cells are important components of the innate immune system. It has been investigated that MDV efficiently infects NK cells (Bertzbach et al., 2019) and macrophages (Barrow et al., 2003). However, how MDV infection of macrophages and NK cells influences the innate and subsequent adaptive immune response remains further to be investigated.

ILTV is classified in the genus *Iltovirus*, subfamily *Alphaherpesvirinae* within *Herpesviridae* family (Gowthaman et al., 2020). The double strand DNA genome of ILTV is about 150kb, containing short and long unique regions (U_S_, U_L_), and inverted repeats (IR, TR) flanking the U_S_ region (Fuchs et al., 2007; Gowthaman et al., 2020). ILTV mainly infects the upper respiratory tract of chickens and causes different clinical symptoms depending on the virulence of the infected strains. The mortality rates caused by ILTV infection vary from 0 to more than 70% (Ou and Giambrone, 2012). Live attenuated vaccines have been applied to prevent ILTV infection and proved to be efficacious (Garcia and Zavala, 2019). However, similar to other live virus vaccines, vaccination with attenuated ILTV strains also faces the risk caused by the remaining virulence.

ILTV infection destroys the respiratory epithelium and induces the infiltration of inflammatory cells (Gowthaman et al., 2020). Inflammatory responses are essential in the innate immune response against ILTV infection. An *in vitro* analysis reported that ILTV infection upregulated many genes associated with inflammatory response, including IL-6 and IL-8 (Coppo et al., 2013). Further studies have identified glycoprotein G (gG), a protein conserved among *alphaherpesviruses*, as a virulence factor that can inhibit cytokine and chemokine transcriptions, thus interfering with the antiviral inflammatory responses (Coppo et al., 2018). gG also influences the composition of inflammatory cell infiltration in the tracheal mucosa (Coppo et al., 2018). However, more details about how ILTV evades host innate immune responses remain unknown.

FPV belongs to the *Avipoxvirus* genus, *Poxviridae* family. As a prototypic species of poxviruses, FPV is a large enveloped virus containing double-stranded DNA genome (Giotis and Skinner, 2019). FPV infects domestic chickens and gallinaceous birds, causing diseases mainly presented as diphtheritic and cutaneous forms (Giotis and Skinner, 2019). Vaccination has been applied to prevent FPV infection (Bhanuprakash et al., 2012). However, in regions where biting insects are prevalent, fowl pox still threats the poultry industry.

Like the other poxvirus, FPV has been extensively used as vaccine vectors for different poultry and human diseases (Skinner et al., 2005). FPV infection inhibits host innate immune response, especially IFN-I response. It has been investigated that *fpv012* and *fpv184* genes of FPV block the expression of IFN-I (Laidlaw et al., 2013; Giotis et al., 2020), while *fpv014* gene contributes to the evasion of FPV from the antiviral effect of IFN-I (Buttigieg et al., 2013).

### ChcGAS

cGAS, which belongs to the nucleotidyltransferase family (Sun et al., 2013), expresses and functions in multiple species (Wang et al., 2015; Li et al., 2020; Qiao et al., 2021). cGAS binds DNA through both the C-terminal NTase domain and N-terminal domain in a sequence-independent manner (Civril et al., 2013; Gao et al., 2013; Kranzusch et al., 2013), and both foreign and host DNA can activate cGAS. cGAS binding to dsDNA undergoes conformational changes and then initiates the synthesis of the nucleotide 2'3'-cGAMP from ATP and GTP (Ablasser et al., 2013; Li et al., 2013a; Andreeva et al., 2017; Hall et al., 2017; Du and Chen, 2018). 2'3'-cGAMP is a second messenger bound by the Stimulator of Interferon Genes (STING; Ablasser et al., 2013; Zhang et al., 2013), a signaling adapter at the endoplasmic reticulum (ER). The binding of cGAMP activates STING, followed by translocating to the Golgi and activating downstream pathways to induce the IFN-I and inflammatory cytokine response (Ouyang et al., 2012; Shang et al., 2012, 2019; Zhang et al., 2019). Infections with cGAS-deficient cells and mice have indicated that the cGAS-STING pathway is indispensable for host immune response against DNA virus infections (Li et al., 2013b; Schoggins et al., 2014).

cGAS also functions in chickens (Vitak et al., 2016; Li et al., 2020; Neerukonda and Katneni, 2020). There is 438 aa in Chicken cGAS (chcGAS), and its predicted structure is highly similar to that of human cGAS. The homology of chcGAS with human cGAS is 48.5% (Li et al., 2020). The chcGAS mRNAs are expressed in various chicken tissues, such as the bursa of Fabricius, thymus, spleen and lung, with the highest in bone marrow (Wang et al., 2020). Subcellular localization assay has indicated that chcGAS does not localize in the mitochondria, lysosomes or Golgi apparatus, while it distributes in the cytosol and is partially co-localized with the endoplasmic reticulum (ER; Wang et al., 2020).

chcGAS responses to exogenous DNAs derived from virus infection or dsDNA transfection, and endogenous dsDNA upon DNA damages (Li et al., 2020). Overexpression of chcGAS induced IFN-β and IL-1β mRNA expression in a way dependent on chSTING, chTBK1 and chIRF7 (Wang et al., 2020). Transfection of poly(dA:dT) and HS-DNA potently promoted the induction of IFN-β and IL-1β by chcGAS (Li et al., 2020). Knockout of chcGAS reduced the IFN-β and IL-1β mRNA expression induced by DNA virus infections, such as HSV-1 and FAdV-4, and subsequently enhanced virus replication (Wang et al., 2020).

### ChSTING

STING has been identified as an essential adaptor that facilitates innate immune signaling against DNA and DNA virus infections (Ishikawa and Barber, 2008; Zhong et al., 2008). Further studies indicated that STING functions as an indispensable adaptor downstream of multiple DNA sensors (Unterholzner et al., 2010; Zhang et al., 2011; Ferguson et al., 2012; Dunphy et al., 2018; Wang et al., 2019; Li et al., 2019b).

There is 379 aa in chicken STING (chSTING). As predicted, chSTING contains the TMEM173 domain (aa 50–342), which is highly conserved (Ran et al., 2018; Li et al., 2020). Sequence alignment showed that chSTING has about 43% identities in aa to human STING (Cheng et al., 2015; Li et al., 2020). chSTING mRNAs are broadly expressed in chicken tissues, especially with the highest levels in the spleen (Cheng et al., 2015). Similar to its human homolog, chSTING is also predominantly localized in the outer membrane of the ER as well as the mitochondrial membrane (Cheng et al., 2015).

chSTING has an essential role in initiating innate immune responses against virus infections in chickens. Overexpression of chSTING in DF-1 cells activated IRF-7 and NF-κB to trigger the expression of IFN-β as well as IFN stimulated genes (ISGs; Cheng et al., 2015). Pro-inflammatory cytokine mRNAs are also upregulated upon chSTING overexpression (Cheng et al., 2015). Moreover, transfection of chSTING into DF-1 cells suppressed AIV and NDV replications, while knockdown of endogenous chSTING inhibited poly(dA:dT)-, and poly(I:C)-triggered IFN-β induction (Cheng et al., 2015). Further studies have shown that chSTING is an essential adaptor in innate immune responses. chSTING mediates the IFN-stimulating signals triggered by different DNA and RNA sensors, including chMDA5 (Cheng et al., 2015), chcGAS (Li et al., 2020), chDDX3X (Niu et al., 2019a), and chDDX41 (Cheng et al., 2017). Endogenous Co-IP and LC–MS/MS suggested that, as in mammalian cells, chSTING interacted with chTBK1 to stimulate IFN-I responses (Cheng et al., 2018).

However, many important issues have not been clarified, such as the features of dsDNA bound by chcGAS, the structures, and mechanisms employed by chcGAS, and chSTING to deliver signals and the regulation of chcGAS-STING pathways.

### ChDDX41

RNA helicases are another group of sensors that participate in DNA-induced IFN-I response. RNA helicases comprise two subgroups: the DEAD-box helicases (DDX) and the DEAH-box helicases (DHX; Briard et al., 2020).

DDX41, which belongs to the DEXDc family of helicases, has been identified as a DNA sensor in DCs in humans and mice (Zhang et al., 2011). chDDX41 has also been cloned and analyzed (Cheng et al., 2017). Sequence analysis has indicated 1,815bp in chDDX41, encoding for 604 aa residues, which shares 93.7% identity with human DDX41 (Cheng et al., 2017). chDDX41 distributes widely in multiple tissues, especially with the highest expression in the crop (Cheng et al., 2017). Although the other members of the DExD family have been identified as IFN-stimulated genes, the expression of chDDX41 is not upregulated by IFN-β. Like human DDX41, chDDX41 is essential for the IFN-β induction in response to dsDNA poly(dA:dT) transfection and DNA virus infection (Cheng et al., 2017).

Human DHX36 and DHX9, members of the DHX helicases, besides their RNA-sensing function, also have a role in sensing CpG DNA and inducing IFN-I and pro-inflammatory cytokine responses *via* IRF7 and NF-κB pathways (Kim et al., 2010). DHX9 binds CpG-B by the DUF Domain, while DHX36 binds CpG-A through the DEAH Domain (Kim et al., 2010). Knockdown of DHX36 or DHX9 potently reduced the IFN and pro-inflammatory cytokine expressions in pDCs infected with a DNA virus while had no impact on an RNA virus infection (Kim et al., 2010).

An evolutionary analysis of the DEAD-box helicase family has identified that DHX9 is genetically deficient in chickens while DHX36 is conserved (Sato et al., 2015). Thus far, the DNA sensing and antiviral function of DHX36 in chickens remain to be explored.

### ChTLR21

As a group of evolutionarily conserved molecules, Toll-like receptors (TLRs) sense different PAMPs. Of them, Toll-like receptor 9 (TLR9) residues in endosomes for sensing DNA. The structure of TLR9 comprises a Toll/IL-1 receptor (TIR) domain and a leucine-rich repeat (LRR) domain (Briard et al., 2020). Upon binding to unmethylated CpG DNA, TLR9 dimerizes and recruits the adaptor protein MyD88, leading to the IFN-I and inflammatory cytokine responses through the transcription factor NF-κB, IRF7, or IRF1 (Briard et al., 2020).

However, the counterpart of mammalian TLR9 does not exist in the chicken genome. Instead, there is a functional homolog to mammalian TLR9 in the recognition of CpG oligodeoxynucleotides (ODNs), that is, chicken TLR21 (chTLR21), a protein of 972 aa residues (Brownlie et al., 2009; Keestra et al., 2010). It has been predicted that chTLR21 has three domains: a cytoplasmic (TIR) domain, a single transmembrane region, and an extracellular domain containing 27 LRRs with cysteine-rich capping structures at both ends (Keestra et al., 2010). Compared to TLR21 of other species, chTLR21 has high similarities with TLR21 of *Xenopus tropicalis* (61%) and *Takifugu rubripes* (57%) and appears more distantly to murine TLR13 (47%; Chuang et al., 2020). As for hTLR9, there is only 38% similarity in aa between chTLR21 and hTLR9, while two aa residues, which are essential for the interaction between CpG DNA and TLR9 (Asp534 and Tyr536), appear to be conserved in chTLR21 (Rutz et al., 2004; Wei et al., 2009). Cellular distribution analysis using confocal laser microscopy on transfected HEK293 cells has suggested that the co-expressed chTLR21-FLAG and hTLR9 have similar localization profiles inside the cells (Chuang et al., 2020). chTLR21 is mainly expressed in immune-relevant tissues. The bursa of the Fabricius and spleen express the highest level of chTLR21, while the lung and thymus have a modest expression (Chuang et al., 2020). chTLR21 expression could be induced by stimulation with CpG-ODN. Inoculation of class B CpG ODN subcutaneously potently upregulated the expression of TLR21, interleukin (IL)-1β, and interferon (IFN)-γ mRNA in the blood cells (Chrzastek and Wieliczko, 2014). Another study conducted *in vitro* indicated that the mRNA expression levels of TLR21 and IFN-γ were substantially increased by Class B CpG ODN stimulation at 1h post-stimulation in a concentration-dependent manner, while this phenomenon only exited in HG cells isolated from young birds (Chrzastek et al., 2014). chTLR21 expression could also be induced by poly (I:C), a dsRNA mimics stimulating TLR3 signaling pathways, in DF-1 cells (Jang and Song, 2020).

Although chTLR21 acts as a functional homolog of mammalian TLR9, there are differences between chTLR21 and hTLR9 in ligand specificities. For example, hTLR9 prefers the CpG-DNA hexameric motifs GTCGTT, while various CpG-ODNs hexamer motifs can activate chTLR21 (Keestra et al., 2010; Chuang et al., 2020). A recent study further revealed that CpG-ODNs with lengths between 15 and 31 nucleotides and different spaces between CpG-hexamer motifs could be sensed by chTLR21 (Chuang et al., 2020).

Stimulation of HEK293 cells in which chTLR21 was transfected with CpG-ODNs resulted in NF-κB activation and subsequently IL-1β and IL-6 mRNA expressions. Using RNA interference (RNAi) technology has confirmed that MyD88 is dependent on TLR21 in response to CpG-ODN in HD11 macrophages (Keestra et al., 2010; Chuang et al., 2020). However, the exact molecular mechanism of chTLR21 activation and signal transduction remains to be explored further.

It has been reported that chTLR21 has been involved in host antiviral responses recently. CpG-ODN, the TLR21 agonist, significantly increased the IL-2, IFN-γ, IL-1, and IL-4 levels in the peripheral blood mononuclear cells (PBMCs) of chickens which responded to IBDV infection and greatly reduced the mortality rate of vvIBDV-infected chicks compared with the birds in the control group (Sachan et al., 2019). Treating the 9–11-day-old SPF embryonated chicken eggs (ECEs) with CpG-ODN induced ISGs, IL-1β, and IFN-γ *viz*. OAS and iNOS genes in CAM. Furthermore, co-inoculation of TLR21 ligand CpG-ODN and avian IBV significantly reduced virus titers (Sharma et al., 2020). Besides, goose TLR21, which has homology to chTLR21, has been reported to have a role in sensing invasive DNA viruses. TLR21, IFN-I, pro-inflammatory cytokines (IL-6 and IL-1β) were potently induced in PBMCs challenged with agonist ODN2006 and new type gosling viral enteritis virus (NGVEV; Qi et al., 2016). TLR21 also has a role in duck immune response against DNA and DNA viruses. Stimulation of DEFs transfected with duTLR21 with CpG-ODN activated NF-κB, followed by the increased expression of the IFN-α, IL-6, and IL-1β mRNA, whereas knockdown of duTLR21 impaired the expression of these genes (Cheng et al., 2019; Chuang et al., 2020). Furthermore, an *in vitro* study has indicated that ectopic expression of duTLR21 blocks the duck plague virus (DPV) replication, while knockdown of duTLR21 efficiently facilitates DPV replication (Cheng et al., 2019).

## Chicken DNA Virus’ Evasion from DNA Sensor-Induced Antiviral Response

**Figure 1:**
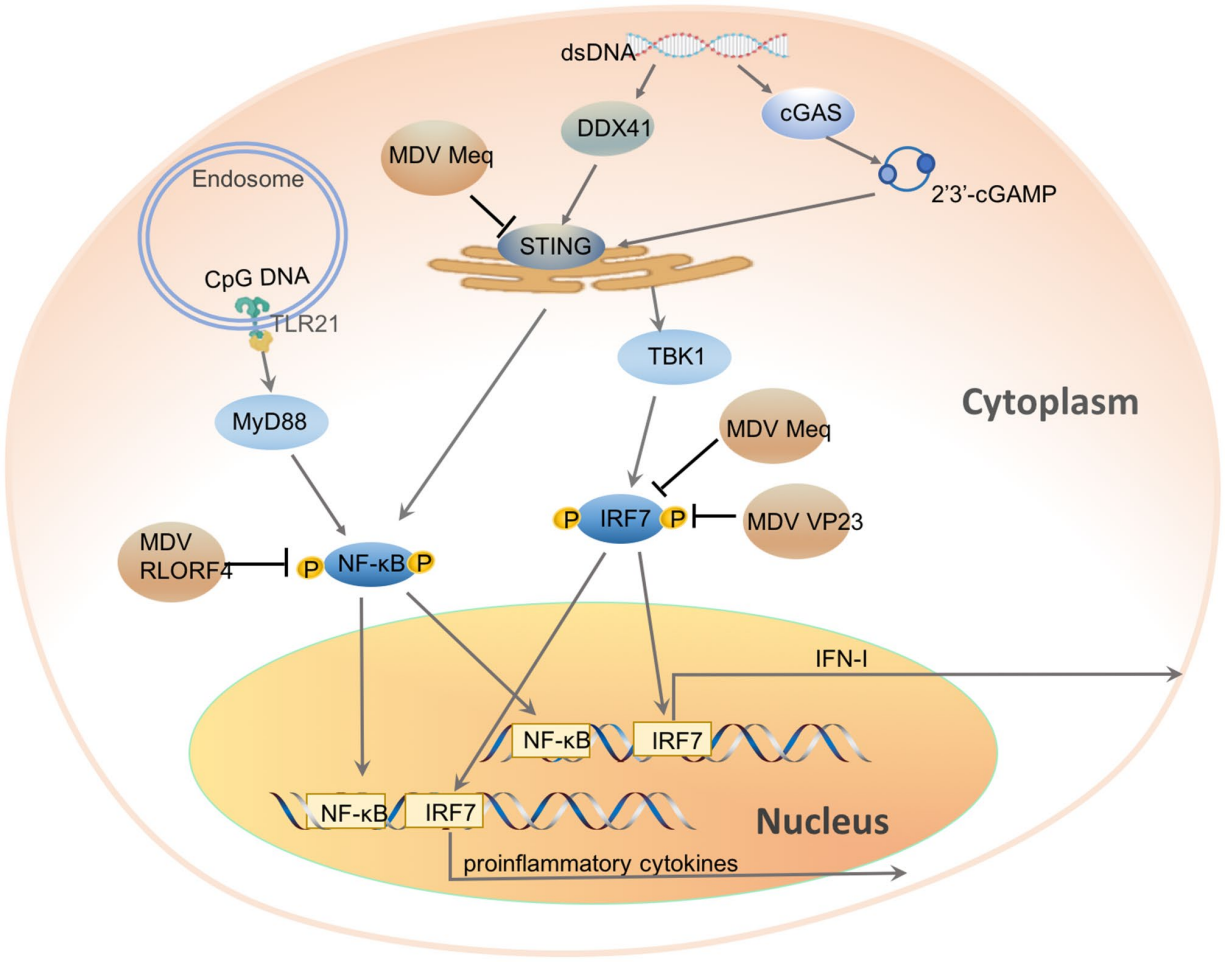
Chicken DNA viruses’ evasion of DNA sensor-induced antiviral innate immunity. cGAS and DDX41 localize in the cytoplasm and sense cytosolic dsDNA directly, DNA recognition induces cGAS and DDX41 activation. Upon activation, cGAS catalyzes the synthesis of the nucleotide 2'3'-cGAMP from ATP and GTP, which will be bound by STING and induce STING activation, while DDX41 interacts and activates STING directly. STING activation recruits and activates TBK1, followed by phosphorylation and nuclear translocation of transcription factor IRF7, which initiates transcription of IFN-I and pro-inflammatory cytokines. STING also activates NF-κB. TLR21, which resides in the endosome, senses CpG DNA and activates NF-κB through adaptor protein MyD88. MDV encoded proteins Meq interacts with STING and IRF7, VP23 interacts with IRF7, while RLORF4 binds the endogenous NF-κB.

Chicken DNA viruses have evolved various mechanisms to inhibit the chcGAS-STING signaling pathway to achieve a successful infection. MDV is a highly pathogenic and oncogenic chicken herpesvirus. MDV infection in chickens induces immunosuppression and fatal T cell lymphomas. To achieve a persistent infection, MDV suppresses host innate immune responses. Knock-down of cGAS or STING in cells has shown that cGAS-STING pathway has an important role in the inducing IFN-response against MDV infection (Gao et al., 2019). Further studies have revealed that multiple MDV proteins inhibit IFN-response through different mechanisms (Figure 1; Gao et al., 2019; Li et al., 2019a; Liu et al., 2019). VP23 is an integral capsid protein of herpesvirus, essential for capsid assembly and viral replication (Kut and Rasschaert, 2004). Besides, VP23 is also essential in regulating host antiviral immunity *via* inhibiting IFN-I induction activated by cGAS-STING. Overexpression of VP23 potently attenuated the induction of IFN-I upon viral infection and enhanced viral replication in chicken fibroblast (DF-1) and chicken macrophage (HD11) cell lines, while VP23 knockdown during MDV infection promoted the IFN-response and inhibited MDV replication (Gao et al., 2019). Dual-luciferase reporter assay has suggested that VP23 inhibits the activation of IRF7 while does not affect NF-κB. By interacting with the same region of IRF7 as TBK1, VP23 also disrupted the TBK1-IRF7 association, thereby preventing IRF7 activation and inhibiting IFN-induction in VP23-transfected cells (Gao et al., 2019). Meq is a major oncoprotein of MDV and has an essential role in counteracting host innate immune response (Li et al., 2019a). Meq can prevent the associations of STING-TBK1 and STING-IRF7 by interacting with STING and IRF7, thereby suppressing IRF7 activation and IFN-β response. Moreover, overexpression of Meq selectively suppresses the expression of IFN-I and downstream antiviral genes upon DNA virus infection and cytosolic dsDNA stimulation. In contrast, MDV with Meq-deficiency significantly induced higher levels of IFN-β and downstream IFN-stimulated genes, resulting in attenuated viral replication and transformation in CEFs and chickens. Being different from VP23 and Meq, MDV RLORF4 selectively inhibits cGAS-STING-mediated NF-κB activation but not IRF7. RLORF4 is a 142-amino-acid protein encoded by a single transcript in wild-type strains. Attenuation of MDV *via* serial passages *in vitro* has indicated that RLORF4 is essential for MDV pathogensis. Deletion of RLORF4 from virulent MDV significantly inhibited viral pathogenicity *in vivo* (Jarosinski et al., 2005, 2015; Jarosinski and Schat, 2007; Vega-Rodriguez et al., 2019). Further studies have indicated that ectopically expressed RLORF4 suppresses the activation of IFN-β promoter stimulated by cGAS and STING, while knockout of RLORF4 from the MDV genome enhances IL-6 and IFN-β productions *in vivo* and *in vitro*. By interacting with NF-κB subunits p65 and p50, RLORF4 could prevent the nuclear translocation of p65 and p50 induced by interferon-stimulatory DNA and TNF-α, thereby inhibiting IFN-β responses (Liu et al., 2019). However, as an oncogenic herpesvirus, MDV encodes multiple proteins which have different roles in viral replications (Davidson, 2020). Whether other proteins have effects on the DNA sensing pathway remains to be investigated.

Nevertheless, how other chicken DNA viruses, such as chicken infectious anemia virus (CIAV), fowl adenovirus (FAdV), and infectious laryngotracheitis virus (ILTV), evade cGAS-STING mediated antiviral signaling is still obscure.

However, there is limited information on how chicken DNA viruses evade other DNA sensors to induce antiviral innate immune responses, which requires further investigation.

## Future Perspectives

The ability of viruses to modulate host antiviral innate immunity is crucial for successful infections. Viruses can adopt multiple strategies to escape host immune surveillance, such as avoiding the exposure of the genome to DNA sensors, inhibiting the synthesis of the second messenger, and blocking the activation of signaling adaptors. Great gaps still exist in understanding how chicken DNA viruses inhibit host innate immunity and get infections, which blocks the design of a new generation of effective vaccines.

## Author Contributions

YW and LG conceptualized the mini-review topic. LG and YW contributed to writing the review. LG, SZ, and YW contributed to editing the review. All authors contributed to the article and approved the submitted version.

## Funding

his work was supported by grants from the National Natural Science Foundation of China (# 32072850) and Earmarked Fund for Modern Agro-Industry Technology Research System (#CARS-40), China.

## Conflict of Interest

The authors declare that the research was conducted in the absence of any commercial or financial relationships that could be construed as a potential conflict of interest.

## Publisher’s Note

All claims expressed in this article are solely those of the authors and do not necessarily represent those of their affiliated organizations, or those of the publisher, the editors and the reviewers. Any product that may be evaluated in this article, or claim that may be made by its manufacturer, is not guaranteed or endorsed by the publisher.
